# Book Reviews

**Published:** 1820-09

**Authors:** 


### CRITICAL ANALYSIS

OF

### RECENT PUBLICATIONS, IN THE DIFFERENT BRANCHES OF MEDICINE AND SURGERY.

"I would have men know, that though I reprehend the easie passing over of the causes of things, "by ascribing them to secret and hidden vertues and properties; (for this hath arrested and laid "asleepe all true enquiry and indications;) yet I doe not understand but that, in the practical "part of knowledge, much will be left to experience and probation, whereunto indication cannot "so fully reach: and this not only in *specie*, but in *individuo*. Yet it was well said, *Vere scire* "*esse per causas scire*."—BACON.

*A Treatise on Inflammation of the Mucous Membrane of the Lungs. To which is prefixed, an Experimental Enquiry respecting the Contractile Power of the Blood-vessels, and the Nature of Inflammation.* By CHARLES HASTINGS, M.D. Physician to the Worcester Infirmary; late President of the Royal Medical Society of Edinburgh, &c. 8vo. pp. 420. Underwoods, London, 1820.

WE had not perused many sentences of the Preface, before we felt disposed to entertain some degree of prejudice in favour of this work; the author so immediately indicates the accurate and judicious views from which the greater part of it has originated. He commences with stating, that "it will be admitted that the effects of inflammation vary considerably in the different textures of the human body, and that it is of the first importance accurately to distinguish the morbid changes which occur in these several textures when inflamed. But so intricate is the animal economy, that a minute acquaintance with diseased structure is not easily obtained; and the imperfection of our pathological knowledge, in this respect, is universally felt and regretted. Unfortunately, as far as regards inflammatory diseases of the pulmonic system, the truth of the above observations is but too obvious; most authors being content to treat of inflammation of the serous, cellular, and mucous, membrane of the lungs, under one common title, —pneumonia."

Before we arrive at the development of those views, we have to consider some observations on the contractility of the arteries, and on the nature of inflammation in general, which are given as elucidatory preliminaries to the more express subject of the work.

As the blood-vessels are the agents by which most of the phenomena of inflammation are produced, Dr. Hastings considers that a rational pathology of inflammation must rest on a previous knowledge of their healthy functions; and, with the view of supplying this knowledge, he enters into a discussion on the contractility of the blood-vessels, having previously endeavoured to settle the acceptation of the terms 'irritability,' 'sensible organic contractility,' 'insensible organic contractility,' and 'tonicity,' as they are employed in this work. "Irritability," he says, "is here used to express that vital power in any living part, by which it contracts or shortens its fibres when touched by a stimulus." The other faculties above enumerated, the author seems to regard as modifications of this power, differing from it only in the extent to which they are manifested.

The author next discusses in a rapid manner the question respecting the part performed by the heart in the circulation of the blood. "Writers," he says, "have always had recourse to both a negative and positive mode of proving a contractile power in the blood-vessels. They have shown that the heart is not adequate to the circulation of the blood; and, by experiments on living animals, have demonstrated a vital contractility in the arteries and veins." For our own parts, we wish the author had mentioned the names of the writers who have shown that the heart is not adequate to the circulation of the blood; for we are not acquainted with one who has done this, though we know that there have been many who have asserted it; but any man who does not place his assertions on such a footing, by valid arguments, as may make them appear entitled to belief, must expect to find his appeal to the judgment of others met with reserve, if not with contempt. Such an appeal is, indeed, an insult to the common sense of mankind. The existence of the faculty of vital contractility in the arteries and veins, does not prove that such a contractility has any thing to do with the ordinary circulation of the blood; for the demonstration of this contractility has been made only under preternatural or extraordinary circumstances, and mostly by means of "experiments on living animals," in which they are submitted to the influence of various preternatural or inordinate agents.

In order to calculate whether the impetus with which the blood is thrown from the left ventricle into the aorta is sufficient to distribute it through all the vessels it has to pass, before it returns by the veins to the right auricle, it is necessary, Dr. Hastings properly observes, to assign the precise force with which the blood is impelled into the great artery, and also the exact momentum any given column of blood should receive at the heart, to enable it to circulate with the necessary celerity in a minute capillary tube, unassisted by any contraction of the blood-vessel. But he admits that "even an imperfect knowledge of either of these necessary steps in the investigation is not to be obtained; for, among those who have endeavoured to ascertain the sum of the heart's action, there is such glaring contradiction, that no reliance can be placed on their calculations." Who, then, has "shown that the heart is not adequate to the circulation of the blood"? "Dr. Whytt's opinions on this subject," the author continues to state, "were founded on Dr. Hales' experiments; and he believed that the real remaining force of a globule of blood, when arrived at a red capillary vessel, was not equal to its own weight. At any rate, the motion of the blood, under such circumstances, must be very slow." If this conclusion were not expressed in so serious a manner, we should have suspected that the author intended to trifle with this subject: it is such a strange mockery of reasoning to form such a conclusion on the *belief* of a man, whose belief was founded on experiments on which "no reliance can be placed."

The greater part of these calculators went to work with wrong principles. They imagined a globule of blood setting out from the heart with a certain momentum, which it was obliged to dispose of, in its journey, to every thing it encountered, and which was yet to carry it on without its receiving any new impulse; which would certainly be true, if the arteries emptied themselves previously to another contraction of the heart. But, as this is not the case, and the arteries are always comparatively full of blood, it is evident that every particle of blood in them must receive a new impulse on every contraction of the left ventricle; just as the impulse given to one end of a pole is experienced at the opposite extremity, in a degree approaching more or less to equality according to the elasticity of the substance of which it is constituted, and the bodies in contact with it in its course qualified to carry off its momentum.

The author, in continuation from our last quotation, says, "But, so far from finding the motion of the globules very slow in the minute vessels, which must necessarily happen if the heart were the sole agent in the circulation, actual experiments demonstrate that these globules glide along with considerable rapidity; with so great rapidity, indeed, that, when magnified by the microscope, the eye can scarcely follow them." If the motion of the globules of blood in the small vessels is rapid, and if it must be very slow were the heart the sole agent in the circulation, why then there is nothing more to be said about the matter, but that the arteries, or something else beside the heart, must assist in effecting the circulation. But we acknowledge none of these assumptions of necessity; and the author himself, in the next page, withdraws his confidence in them, by saying, "in every rational investigation wherein it is impossible to arrive at any positive evidence, we should incline to that side of the question which appears most consistent with facts. In this view of the subject, the idea of the impulse given to the blood by the ventricle being the sole cause of its motion, to say the least, seems very questionable." Here we agree with the author, and shall therefore consider the results of the experiments which he adduces in support of the doctrine of the active agency of the arteries. He first, however, argues for the irritability of the arteries, which no one now seems to deny, using the term irritability in the sense in which it is done by the author. Certainly, neither Dr. PARRY nor Dr. CHARLES PARRY has denied them this property: they indeed expressly admit it, and intentionally adduce examples of it in their works on this subject; though the former thought proper to distinguish the contractility of the arteries from that of the muscles, and appropriated to it a different appellation,—that of *tonicity*: whether right or wrong, is quite adventitious to the points of dispute.

But, admitting that the arteries are possessed of irritability, it is with reason doubted whether this irritability is called into action, so as to produce alternate contraction and dilatation of the vessels, in the ordinary or healthy state of the system. Dr. Hastings endeavours to prove the affirmative; but his only arguments are the results of experiments in which the artery was submitted to preternatural influence. We allow that it appears less rational to suppose that contraction and dilatation are exerted only on such occasions, than that it is ordinarily exerted, but in a manner not well evident to the senses, unless it be urged by preternatural stimuli; but this is only, at the best, a rational *conjecture*: no one has pretended to have seen the arteries contract and dilate, in a manner corresponding with the action of the heart, in the healthy state. The argument, often used, that we can feel the artery contract and dilate, when we feel the *pulse* for instance, is shown by the Drs. Parry to be invalid: it is the momentum communicated to the artery by the blood, and the elongation of the artery, that produce the sensation we then experience. It appears that more or less contraction of the arteries was produced by various stimulants, mechanical as well as chemical, in all the experiments of Dr. Hastings; which is remarkable; for HALLER, on speaking of the contraction of the arteries, says, "quam neque sponte agentem unquam viderim, neque irritatio mechanica suscitet." Dr. JONES says, he never but once saw a distinct contraction produced by a mechanical stimulus; and LEEUWENHOEK, that he never saw it in any instance. Dr. Hastings states, moreover, that he has seen even alternate contraction and dilatation in exposed arteries. No previous enquirer on this subject has ever noticed this: and we cannot help suspecting that Dr. Hastings may have mistaken the change of situation suffered by the artery on every stroke of the heart, for dilatation of the vessel. The author argues in favour of the ordinary active contraction and dilatation of the arteries, from the phenomena sometimes observed in disease; but these arguments can never prove what is wished for: though we must allow that they render it probable, very probable; but this is all. The following is an abstract of the results of the author's experiments on the irritability of the smaller or capillary arteries, according to his own statement.

The application of a stimulus often quickens the circulation in the small vessels; and, when a small artery or vein is touched by a stimulating substance, a contraction is often produced, so as to be visible by the help of the microscope. This contraction sometimes proceeds to such an extent as to prevent the free passage of the blood; and in this case it does not accumulate, but takes a retrograde course. A stimulus, too, often produces a quickened motion of the blood and contraction of the vessels, in the first instance; but, after it has been applied for some time, dilatation of the vessels and a slower movement of the blood follow. When the vessels, however, are dilated by the action of one stimulus, some other stimulus will often produce contractions. Thus, the application of water heated considerably above the temperature of the animal, often occasions contraction of the vessels and acceleration of the motion of the blood; but, after a certain time, dilatation and retarded circulation ensue. Ice generally produces a contraction of these dilated vessels, and restores the velocity of the circulation. Ice, kept in contact with the web of a frog's foot, produces, in the first instance, a contraction of the capillaries, and increases the motion of the blood; but, after a certain period, if the application be continued, the vessels become dilated, and the motion of the blood is more languid: a temperature of 80° Fah. however, or the oil of turpentine, again excites the capillaries to contract, and the circulation is restored to its natural state.

"These experiments," says Dr. Hastings, in allusion to those just noticed, "were principally made on the web of the frog's foot;" and, as far as his details go, they were *universally* made on this part. How far the results of them are applicable to warm-blooded animals then remains to be decided; for the diversity in the structure and functions of the two classes is too great to permit them to be applied without reserve. We have one more remark to make, and then we quit this subject. Dr. Hastings speaks of visible contraction and dilatation of those capillaries of the frog's foot in which he could also discern the globules of blood circulating: now we never could distinctly see these vessels at all, they are so pellucid, much less perceive them contract and dilate. The only thing we have been able to see, with the aid of an excellent microscope, is the fluid running through them.

The results of some experiments on the veins of warm-blooded animals are also stated, in proof of the contractility of these vessels.

These premises lead to some considerations on the nature of inflammation. "But," says the author, "as some of the phenomena of this disease depend upon the intimate connection between the sanguiferous and nervous systems, some facts on this subject shall be first briefly stated."

The *fact* first mentioned is, that "it is now an established principle that the action of the heart and blood-vessels is independant of the nervous system. (Dr. Wilson Philip *on the Vital Functions.*) This is abundantly proved by facts. If we take away the spinal cord and brain, the circulation continues for some time in full force. If we remove the heart from the body, it contracts with vigour and alacrity." This is setting up an oligarchy in physiology with a vengeance! What Dr. Hastings and a few others believe, is to be considered "an established principle:" and then for the *proofs* of it; the same arguments might be used to *prove* that the peristaltic motions of the intestines, the secretions, assimilation, nutrition, and the rest of the organic functions, if there be any others, are independant of the nervous system; for they will proceed without the brain and spinal marrow. The heart, Dr. Hastings says, will contract with vigour when removed from the body: and so will the intestines: but these facts do not show that the actions in question are not dependant on nervous influence. We know not how long an organ deriving nerves from the ganglionic system may retain its nervous power, when separated from connection with the centre of this system.

The notions advanced by Dr. Hastings, just mentioned, are chiefly founded on the assumption that the brain and spinal marrow are the only sources of nervous influence; than which nothing is further from being proved: whilst a multitude of phenomena render it probable that the ganglionic system has a distinct power, independant of those organs, which Haller, and those who now profess his doctrine, have not recognized, or have refused to admit, though unable either to disprove it, or to prove what they very complacently advance as a *fact.* It is a curious thing to mark the qualifications of some of the gentlemen who have capered about over this field. Some of them, indeed, seem to have been ignorant of the grossest parts of anatomy: they have talked of the removal of the axillary plexus of nerves from a dog's fore-limb, as of the total interruption of nervous influence to the limb; the mass of ganglionic nerves which twines about, and is intimately blended with, the arteries of the part, is quite neglected; and then, all the functions which have been manifested in the limb, after this removal, are said to be independant of nervous influence.

But, though the ordinary action of the heart and arteries is considered by Dr. Hastings to be independant of the nervous system, he nevertheless says, "certain states of the nervous system are known to produce effects on the action of the heart. Thus, the passions affect the sanguiferous system; spirits of wine applied to the brain quickens the action of the heart; tobacco applied to this organ, first increases, and then diminishes, its functions." It is worth noticing here, that the active contraction and dilatation of the arteries in a morbid or inordinate state, is brought forward by the author, in proof of their contraction and dilatation in the healthy state. Why did he not see that, by the same rule of argument, it might be said that the action of the heart and arteries is always influenced by the nervous system. Not that we think such an argument good for much; but he should have preserved some such consistency in his disquisitions. We should have been glad to have been informed, too, how a substance, as tobacco, applied to a portion of the nervous system, can diminish the functions of a part whose actions are independant of this system.

We proceed to the author's observations on inflammation. We have already stated the results of several of his experiments on the influence of stimuli on the capillaries, on which his opinions are founded. But as the chief of his observations, as well as inferences, on this subject, are similar to those published some years since by Vacca, and so well argued for by Dr. Wilson Philip, in his *Treatise on Fevers,* it is not proper to enter into a detailed examination of them on this occasion. Dr. Hastings has certainly supported with considerable vigour, and with much perspicuity, the doctrine that the vessels of an inflamed part are in a state of comparative debility, in consequence of which they are passively distended by the impulse of the blood transmitted to them. He endeavours also to reconcile this doctrine with the observations dwelt on by those who have maintained that inflamed capillaries act with more vigour, and propel forward their contents more rapidly, than in the healthy state, at the same time that they are inordinately dilated; by attempting to prove that two very different conditions of the vessels have been confounded together, one of which is a state of excitement, absolutely of a different nature from that which constitutes inflammation. Increased velocity of the blood, he says, does occur on the application of certain stimulants to the textures of an animal body, and increased action of the smaller arteries; but the part is paler than natural under these circumstances, instead of presenting the chief characteristic of inflammation,—inordinate redness: it is not until the vitality of the vessels seems to be partially destroyed, by the continued influence of the stimulant, that they become dilated, so as to cause preternatural redness of the part; and then the circulation is always more or less languid, if the blood be not absolutely stagnant. The effects of alternations of diverse stimulants in this state of the vessels, have been described in a former part of this article. Dr. Hastings says, also, that, "if the stimulus which produces the inflammation be of a very acrid nature, debility of the vessels is frequently induced without any previous excitement. The blood in all the smaller vessels becomes very red, circulates very slowly, and in some vessels stagnates." How debility can be immediately produced in vessels by an acrid stimulus, without any previous excitement, we cannot at all comprehend. But such an assumption was necessary, in order to place the fact in question in a point of view consonant with the doctrine the author argues for, and to obviate the objection it obviously presents to this same doctrine. A doctrine that requires such sacrifices to be made to close and sound reasoning, must be regarded with much reserve; though it must at the same time be acknowledged, that this doctrine requires fewer gratuitous assumptions to render it plausible, than that which makes the capillaries to be in a state of active dilatation, and should therefore be preferred to it. The effects of the remedial measures we employ for inflammation are, likewise, more satisfactorily explicable by the doctrine supported by the author, than by that which it is here opposed to.

The second chapter of the work is devoted to an examination of the opinions of preceding writers respecting bronchial inflammation; two of the most important of which, Bayle and Broussais, are not noticed by the author; both of whom, and especially the latter, would have furnished much interesting matter relating to this disease, collected from very extensive observation, under circumstances peculiarly favourable for the investigation of its consequences, and assiduous researches in pathological anatomy. It is a curious circumstance, that most of our writers stop in the middle or latter part of the last century, in their historical accounts of the contributions of foreign nations to medical science; and that amongst those, too, who, as Dr. Hastings has here done, have taken up Pinel's views in nosography, not one, excepting a few of the Dublin physicians, has mentioned this source of the greatest improvements in modern pathology.

After what has been said respecting bronchitis by former authors, but little remained to be advanced respecting it, considered in itself, without relation to other diseases, and in regard to either its history or diagnosis: we could not, therefore, point out much of great importance, distinctly regarded, that is novel in these respects to the well-informed practitioner, in the account given of it by Dr. Hastings; though we must at the same time state, that his account is a very good one, being accurate, perspicuous, and sufficiently comprehensive: it therefore presents what cannot be otherwise attained, without investigating many different sources. Besides this, Dr. Hastings has distinguished the several varieties of this affection, as well as pointed out their relations to some other diseases, with more accuracy than any former author. His *ratio symptomatum*, too, is perspicuous and satisfactory, and in several points original; and the results of his observations in respect to the treatment of the disease, present much that will be interesting and useful to by far the greater proportion of medical practitioners. We shall consider the latter points in a somewhat particular manner, after having given an abstract of Dr. Hastings' history of the several forms of this affection. He makes seven varieties of acute inflammation of the mucous membrane lining the bronchiæ in their distribution through the lungs.

1. The common catarrh, which is the mildest form of the disease, but which often degenerates into an obstinate chronic affection in persons of delicate habits, when it is neglected or ill-treated. The cough then becomes severe; the expectoration copious and resembling pus; the symptoms of phthisis supervene; and dissection after death presents evidence of inflammation of the mucous membrane of the lungs, and often tubercles dispersed in the substance of these organs; the development of which Dr. Hastings attributes to the inflammation of the mucous membrane: a point of doctrine well established by Broussais,* and made by him the source of many highly important inferences.

2. A form of the disease as it occurs especially in old persons and those of phlegmatic habits, especially during sudden changes of weather. There is here no fixed pain in the chest, but a sense of weight and straitness. The respiration is quick, laborious, and accompanied with wheezing: there is cough, at first often dry, afterwards attended with copious expectoration of thick, viscid, opaque mucus, sometimes moulded into the shape of the ramifications of the bronchiæ; almost always great pain across the forehead, and often drowsiness and vertigo. The pulse, in the course of the disease, if not in the first instance, becomes full and hard, though it has not the extreme hardness and vibration of the pulse in pleurisy; and evening paroxysms of restlessness, with flushing of the face, come on.

---

* See our exposition of this part of his doctrine, in vol. xliii. p. 147 *et seq.* of this Journal.

When the progress of the disease is not checked in severe cases, the pulse becomes very quick and much weaker; the lips, face, and fingers under the nails, assume a livid hue; the countenance evinces great distress and anxiety; the extremities become cold, and death takes place, apparently from suffocation, produced by the presence of the fluids collected in the bronchiæ, or want of oxygenation of the blood. In other cases the disease begins to disappear about the sixth or seventh day; and in some instances it degenerates into a chronic form. When the patient dies in the first stage, above mentioned, dissection shows a highly-diseased state of the bronchial membrane, whilst the structure of the lungs is generally not affected.

3. A more acute form, in persons of more strong and plethoric habits, in which there are present signs of more severe local inflammation and re-action of the general system. Even here there is, however, but rarely any fixed pain in the chest: a very distressing sense of straitness, with hurried and laborious breathing, are the most striking of the local symptoms. Wheezing is not so constant here as in the foregoing form of the disease, the secretion not being so copious in general; though it is more likely to accumulate and suffocate the patient. It sometimes terminates fatally as early as the fifth day. It may have, also, the same modes of termination as the forms previously described.

4. An acute affection, to which young children are peculiarly subject, even more speedily fatal than the last variety, but which does not produce symptoms of corresponding severity. It commences as a catarrhal disorder, with wheezing, but no considerable degree of difficulty of breathing; the cough is often but slight; there is but little expectoration; and in many cases but little fever. There is commonly a remarkable pallidity of the countenance. Remissions and exacerbations of the dyspnœa ordinarily occur throughout the disease. There is commonly a livid tinge in the lips, and often in the face. Coma generally supervenes in fatal cases, and here death takes place ordinarily between the fourth and the seventh days.

5. Bronchitis in connection with various cutaneous diseases, as erysipelas, variola, and rubeola; and that which succeeds to the disappearance of some chronic affections of the skin. Some more precise knowledge of the relations of these affections is yet wanted.

6. Bronchitis complicated with affections of the abdominal viscera. The most frequent connection of this kind is with disease of the liver. The bronchitis here commonly assumes the character of the second variety; and Dr. Hastings is disposed to consider it to be dependant on the hepatic affection, which commonly follows the too free use of spirituous liquors; though he takes care to state that the hepatic disease may have no other connection with the bronchitis than that of rendering the membrane particularly disposed to inflammatory action, and then, from the common exciting causes, the inflammation readily takes place.

7. Bronchitis existing with other diseases of the respiratory organs; the most frequent of which is chronic inflammation of the larynx and upper part of the trachea, and sometimes ulceration of these parts, itself induced by inflammation. Another connection of bronchitis is with tumors situate externally to the trachea and pressing on it, as bronchocele.

In his general remarks on the symptoms of bronchitis, the author says there are several of them which are common to inflammation of the bronchial membrane, and to that of the structure of the lungs: amongst these are dyspnœa and cough. There are other symptoms which are peculiar to bronchitis, as the wheezing, which the author considers to depend on a redundance of the secretion from the mucous membrane. This is also the explanation of LAENNEC; whilst Dr. BADHAM, and some others, attribute it, not so satisfactorily, to the thickened state of the bronchial membrane. The occasional increase of the mucous fluids serves, Dr. Hastings thinks, to account for the paroxysms of increased dyspnœa. The pain in the forehead may depend either on an affection of the mucous membrane lining the frontal sinuses, or on obstruction to the due return of blood from the head by means of the congested state of the lungs, and in some instances, perhaps, on the circulation of imperfectly-decarbonized blood through the vessels of the brain. The extreme debility, the livid hue of the face, &c. depend also on the cause last mentioned. This imperfect decarbonization of the blood originates from the accumulated secretions in the bronchiæ preventing the blood brought to the surface of the air-cells being properly acted on by the air. This effect, with the debility and other consequences of it, are ordinarily proportionate to the abundance and accumulation of the secretion; and hence they are most strikingly manifest in the second variety of bronchitis.

After some very good remarks on the diagnosis and prognosis of these affections, the author enters on the consideration of their treatment. The first indication, he properly states, is the removal of the inflammation of the mucous membrane; this, with the secondary indications, are to be fulfilled in the following manner: "To moderate the excitement of the sanguiferous system,—general blood-letting, acidulated and mucilaginous drinks, and abstinence from all stimulating food. To promote expectoration and perspiration,—antimonial and saline medicines. To direct the fluids towards the surface, and relieve the congestion of the debilitated capillaries,—local blood-letting, blisters, and rubefacients."

Blood-letting, the author states, in his particular remarks, is by far the most powerful of the general means for diminishing the excitement of the system; but it is not equally called for in all the varieties of bronchitis. In the first variety it is but seldom necessary; in the second, it should be employed with caution; sometimes we have found the expectoration cease, whilst the difficulty of breathing has augmented after its use: here it is evidently injurious. The abstraction of ten ounces of blood from the arm, early in the disease, the author says, "sometimes mitigates the symptoms; after which it is generally more safe to depend upon an attention to diet, proper expectorants, and local evacuation." But, in the third variety, blood-letting should be boldly employed: from twenty to thirty ounces of blood may be taken from the arm, in severe cases, in the first instance. The course of the disease is so rapid, that no time should be lost in resorting to it. There are but few cases, too, which yield to one blood-letting.

Acute attacks, in general, require the free use of the same remedy; and Dr. Hastings favours its being practised in the jugular vein.

In the combination of abdominal disease and bronchitis, the degree of debility, he remarks, is sometimes such as not to admit a great loss of blood, when the former affection has existed any great length of time; but, if the abdominal and pectoral inflammations are simultaneous effects, blood-letting should be as freely employed as in the third variety.

Vomiting, and antimonials in small doses, are very beneficial in every variety of the disease. Some of the stimulant expectorants, as squills and ipecacuanha, are useful in the latter stages, especially in the second variety. Laxatives may be used with advantage in most cases; but active cathartics, except at the commencement of the attack, are not beneficial. In the second variety, in old persons, they are apt to check the expectoration, and be productive of mischief. Mercurials, exhibited so as to act upon the system, Dr. Hastings says, are not usually beneficial, except in the sixth variety, when the liver is affected; though, he adds, when bronchitis is combined with inflammation of the trachea, and produces symptoms resembling croup, analogy would lead us to employ calomel in frequently-repeated doses. Opium is prejudicial as long as there is much fever; but, when that declines, and irritability of the system and air-passages still prevails, it not unfrequently allays the cough, and calms the patient: but opiates must be employed with great caution, especially in the second variety; for, when the secretion is copious, and the strength much reduced, they interrupt, for a time, the efforts to expectorate, and may thus prove fatal. In combination with calomel, opium may sometimes be exhibited at an earlier period of the disease.

All the varieties of acute bronchitis may terminate in a state of collapse of all the powers of the system, if the remedies employed do not check the progress of the disease. In that event, we must support the strength of the patient, and endeavour to relieve the bronchiæ of the secretions with which they are clogged. Ammonia, the author thinks best calculated to produce these effects, whilst it is at the same time often serviceable in promoting expectoration towards the decline of the disease. We ourselves think very favourably of this medicine, especially in the latter stage of the second variety, as we stated in one of the late Reports on Disease in this Journal.

The local means of most importance, the author adds, are topical blood-letting and blisters. The former measure he inculcates the use of, in all cases where it is necessary to repeat general blood-letting, in combination with the latter measure, and where venesection might seem dangerous. Blisters should not be used until the excitement has been considerably relieved by blood-letting. The tepid bath, and warm fomentations to the chest, are also often serviceable.

The author does not speak of the use of either digitalis or the prussic acid, in this disease; both of which, we think, especially the latter, are very powerful, and consequently very valuable, remedies, when properly employed.

Accounts of several very appropriate cases, with the appearances on dissection in such as terminated fatally, in illustration of the foregoing observations and remarks, terminate this chapter. They are accompanied, too, with elucidatory reflections, that will prove very instructive to the practitioner.

The next chapter is on chronic bronchitis. This is often the consequence of acute bronchitis, and very frequently, as Broussais had already proved, and which Dr. Hastings confirms, it terminates in phthisis, by inducing the development of, or rousing from a dormant state, tubercles in the lungs. Sometimes it simulates phthisis very closely, when no tubercles are found after death. "In some varieties of chronic bronchitis (says the author) there is no difficulty in making the distinction between that disease and tubercular phthisis. Those in whom the disease originated from the inhalation of irritating substances, often continue to cough much, and expectorate copiously for years, without losing any flesh; and, excepting when the affection is more than usually severe, do not complain much of dyspnœa. Besides the absence of these symptoms, so characteristic of tubercular consumption, the peculiar pallidity of the countenance, combined with a slightly-livid tinge on the lips, cannot fail to point out the distinction between the former and the latter disease."

Dr. Hastings distinguishes several varieties of this form of the disease. 1st. As it occurs in persons advanced in life, with a chronic cough, which generally attacks the patient in cold weather, or is then much exasperated. The symptoms do not differ remarkably, except in degree, from those of the first variety of acute bronchitis. 2d. With the characters of tubercular phthisis. 3d. As a termination of acute bronchitis. 4th. As a consequence of cutaneous diseases. 5th. As the result of irritating substances acting on the mucous membrane, as gaseous matters, dust of various kinds, &c. 6th. In connexion with diseases of the abdominal viscera. The most frequent, as in acute bronchitis, is with some chronic disease of the liver.

The particular symptoms and most striking characteristics of those varieties, are considered by the author with much acumen. Many practitioners affect to despise such nice distinctions of the varieties of a disease; but it is the knowledge and appreciation of these that makes the difference between a good and a bad practitioner. General indications for the management of diseases are always in a certain degree vague; they never can prompt such effectual remedial means as particular indications; and they very commonly lead to inert or even worse practices. Every remedy employed under the direction of the former assumes almost the character of a specific. As favouring considerably these indications, the work of Dr. Hastings must be considered an addition of considerable value to medical literature, even did it possess no other merit.

The appearances on dissection in the subjects of chronic bronchitis, are, an injected state of the vessels of the mucous membrane of the lungs, sometimes continuous for a considerable extent, at others in patches; ulceration of the membrane; an accumulation of mucus, or bloody and frothy serum, in the air-cells, sometimes mixed with purulent matter. The morbid appearances are less frequently confined to the mucous membrane than in acute bronchitis; the substance of the lungs is sometimes more solid than natural, from a deposition of coagulable lymph in their structure; sometimes tubercles are present both in the lungs and the pleura, and the latter membrane occasionally presents signs of previous inflammation. The pulmonary vessels are always loaded with blood, and dilated beyond their natural size, sometimes in a varicose manner. The heart is often in a morbid state. Both the auricles, but particularly the right, are often dilated. The right ventricle is also sometimes larger than natural; and the auriculo-ventricular valves are occasionally thickened.

The *ratio symptomatum* of the form of bronchitis last mentioned, is given with much accuracy by Dr. Hastings. The origin of the cough and hectic fever must be readily perceptible. The dyspnœa (beyond that which depends on the state of the mucous membrane itself, and the secretions accumulated in the bronchiæ), is accounted for by the enlarged and varicose state of the pulmonary blood-vessels, which enlargement arises from the partial stagnancy of the blood in the capillaries, from its not undergoing the ordinary changes in the lungs, and, consequently, not sufficiently exciting the organs of the circulation to propel it with proper celerity. The same obstruction leads to enlargement of the cavities of the right side of the heart, especially of the right ventricle. This state of the heart and blood-vessels tends to produce accumulation of serous fluids in various parts of the body, by the derangement of the absorbent functions which thence results. This is a combination of morbid states, which we shall consider a little more particularly after we have noticed what the author says respecting the treatment of chronic bronchitis in its more ordinary states.

He first discusses the propriety of the use of blood-letting, vesicatories, rubefacients, and emetics. His observations on these points contain nothing remarkable, except that he insists much on the superiority of local blood-letting by leeches to venesection, in a great proportion of cases. Digitalis, he says, is useful when the disease assumes the form of catarrhal phthisis, and has a tendency to terminate in dropsy: we think highly of its powers in nearly all the forms of the disease. Squills he designates as particularly useful in old persons of phlegmatic habits. The tincture of meadow-saffron he considers to be a very promising remedy in chronic bronchitis, of which we have lately had abundant proof in the extensive practice of a public dispensary. It is sometimes useful to combine with it ipecacuanha or digitalis. The colchicum removes also the symptoms of chronic pleuritis, and serous effusion into the thoracic cavity, with great approach towards certainty, and much rapidity. The vegetable balsams have not appeared to Dr. Hastings to be productive of "so much benefit as he was led to hope from Dr. Armstrong's report." We have ourselves used them less frequently than heretofore, since the utility of the colchicum has become so evident. The author has derived much benefit from a combination of cicuta and ipecacuanha in several obstinate cases. Cinchona is sometimes very efficacious. Some very remarkable and gratifying instances of the powers of this remedy are related by Morton, where the hectic fever was intense in degree, and the expectoration of puriform matter very abundant. Mercury seems to favour the curative efficacy of some other remedies in certain cases; and is itself an important one in that form of the malady dependant on hepatic disease. Dr. Wilson Philip, for the purpose of lessening the quantity of mercury necessary to be given in these cases, has strongly recommended dandelion to be combined with it: the dandelion must be given in large doses. Opium must be cautiously used even in this form of the disorder; but it is useful for allaying irritation in some cases. The extract of lettuce, recommended by Dr. Duncan, may probably often be preferable. The author has tried the tar-vapour; and the inferences from his experience are, that "it appears that, when the habit of body is irritable, and the inflammation at all active, the symptoms are increased by its use; but, if the disease have been long in a chronic state, and the habit of body be not irritable, relief follows its application." Not one instance of even temporary relief from this measure, in tubercular phthisis, has occurred in his practice.

Some observations on diet, clothing, and exercise, and some illustrative cases, constitute the remainder of this chapter.

Dr. Hastings does not appear to have ever used the prussic acid in the affections we have been considering. This we are somewhat surprised at, considering the extraordinary efficacy of that remedy, and the manner in which it has been recommended to the profession by Dr. Granville.

Although anasarca, consequent on inflammation of the mucous membrane of the lungs, may be well supposed to have been in all times a frequent occurrence, for it is so common, that thirteen instances of it have occurred to our own observation within three months, in the practice of a Dispensary where the constant list of patients varies from fifty to eighty; yet the intimate relation of the two affections was never pointed out until about two years since, when it was stated by Dr. Crampton,* unless Dr. Stoker may be considered to have given a hint of it,† a short time previously. Many cases of this kind are related by former writers; and some possessing remarkable interest are contained in the classic work on Dropsies of Dr. Blackall; but no other had shown, before those just designated, that the dropsy was dependant on the state of the mucous membrane of the lungs as its original cause. That such a relation exists between the two affections, is as satisfactorily evident as almost any law in pathology. Hydrothorax and ascites, of course, often bear the same relation to bronchitis as anasarca.

A very good history of this disease is given by Dr. Hastings, especially with the view of showing the dependance of the dropsy on bronchial disease. He also endeavours to point out the influence of disease of the heart as an intermediate link in

---

* In the *Trans. Assoc. Dublin, Coll. Phys.* vol. ii. p. 263.

† Ibid. vol. i.

this chain of morbid phenomena, in some instances, in the way we mentioned when speaking of the symptomatology of chronic bronchitis. The author's general conclusion is, that "all the dropsical symptoms seem to proceed from the impediment to the motion of the venous blood, which may be attributed to its imperfect flow through the heart, and to the congestion of the pulmonary vessels." Diseases of the liver and digestive organs sometimes act inferior parts in this development of morbid phenomena; but the hepatic disease is much more frequently the primary affection. This is not, however, the only species of dropsy dependant on bronchitis. The inflammation of the mucous membrane is not very unfrequently communicated to the pleura, and, as we noticed in some of the Reports of Diseases during last winter, to other serous membranes; and a more sudden and active species of dropsy is the consequence, which requires a very different mode of treatment from that under express consideration; for in that, depletory measures and counter-irritation, excited on the mucous membranes in some instances, are the means of cure.

The author's remarks on the treatment of dropsy dependant on bronchitis contain nothing that is distinctly remarkable or original. He, as Dr. Crampton had previously done, insists on the indications for the relief of the bronchial affection as the first in the means of cure; though he prefers, in most cases, repeated local blood-letting to venesection. Purgatives are here, he properly remarks, of but very limited utility: diuretics are more beneficial; but very often the most efficacious measure of this kind is blood-letting. Squill, when the bronchitis is not acute, is often very beneficial, especially when combined with digitalis, and, in some cases, the blue pill. The super-tartrate of potash is often of considerable utility in several points of view. He does not seem to have hitherto much used the colchicum in these cases; he only says of it, that it "holds out many inducements to a fair trial of its preparations," in addition to his remarks on its efficacy in relieving chronic bronchitis. The results of the thirteen cases we have already alluded to, admit of inferences highly favourable to its efficacy. Five, which had resisted squills, digitalis, and the rest of the ordinary means, were immediately cured, and three others considerably relieved by it; but in these, and the remainder, too much organic lesion is present to permit any remedy to be fully successful. Seven cases of this species of dropsy are related by Dr. Hastings: four fatal ones, for the purpose of illustrating the nature of the affection, and three of which the termination was in comparative health, to shew the phenomena attending the progress towards this state.

We have given a sufficient exposition of the pathological part of this work, to shew that our prejudice in its favour was well founded; and we have no doubt but that it will be generally referred to by medical practitioners, as the source of much valuable clinical information.

---

*A Report of the Practice of Midwifery, at the Westminster General Dispensary, during* 1818*; including new Classifications of Labours, Abortions, Female Complaints, and the Diseases of Children: with Computations on the Mortality among Lying-in Women and Children, and the probability of Abortion taking place at different Periods of Pregnancy, &c.* By A. B. Granville, M.D. F.R.S. F.L.S. M.R.I. Physician-in-Ordinary to his Royal Highness the Duke of Clarence, Licentiate of the Royal College of Physicians, Physician-Accoucheur to the Westminster General Dispensary, &c. 8vo. pp. 220. 1819.

There is hardly any subject, however dull and barren to the generality of persons, that may not become the source of interesting speculations and various useful inferences to a man of genius. Circumstances apparently the most trivial have given birth to some of the grandest views in science, as well as to many of the finest productions in the *belles-lettres.* It should not, therefore, excite surprise, that the practice in the midwifery department of a Public Dispensary for one year, has here been made the basis of a work, which comprises numerous original statements of remarkable interest to the politician, as well as to the practitioner of medicine. Several good reports of a similar kind had certainly been already produced, but they left much to be regarded by more comprehensive views of the subject in itself, as well as of its relations to others of more or less importance to our social interests. Some of the statistical accounts here adduced by Dr. Granville present the principles of a series of information of considerable value to those who study the regulations of societies for granting annuities on the lives of individuals, or on the probability of that of their existing or expected progeny, of which we shall give a concise statement, at the same time that we point out what is more expressly interesting to the medical practitioner.

An *Introduction* comprises some very pertinent and good remarks on the utility of public dispensaries, and on the relative value of those establishments to hospitals in which sick persons are admitted as residents; a chronological list of the several dispensaries established in the metropolis, with a particular account of the *Westminster General* institution of this kind; and a view of the series of matters treated of in the body of the work.

The first section commences with some considerations on *Parturition,* which furnish the author with an opportunity for proving the great importance and peculiar value of those institutions which afford aid to pregnant women at their own homes in the time of their puerperal confinement. He then adduces some interesting statistical calculations respecting the results of pregnancy, the nature of labours, and the artificial aid required, the mortality, age of the patients, and proportion of children preserved alive at a certain period. Of 640 cases of pregnancy occurring in one year, 627 went the full time: the proportion of boys to girls was as 3 to $2\frac{7}{10}$; the general proportion of still-born children 1 in $31\frac{2}{5}$; but that of boys was 1 in 27, whilst that of girls was only 1 in $37\frac{1}{2}$, a diversity which nearly accords with the calculations of other authors, and is explicable by the greater size of male than female children at the time of birth. Nine women out of the 640 had twins, and produced 14 children alive and at the full time, and four still-born, or before the nine months. The proportion of twin cases was 1 in 71: the children were both males in two cases, both females in four cases, and male and female in three cases.

The labours are divided by the author into *active* and *passive*: by the former term he designates labours "terminated without the slightest interference by nature alone;" by the latter, those in which "nature becomes *passive*, or insufficient, and the assistance either of the *hand* or instruments is absolutely necessary to terminate the labour. This classification of labours is proposed in place of the complicated arrangement of all the existing methods, and is, the author thinks, calculated to answer every useful purpose to the practitioner.

Of the 640 cases already noticed, 619 were active, 515 of which terminated within the first twelve hours. Of the twenty-one passive cases, thirteen received *manual* aid, and eight *instrumental*. The author states that he has found, in general, that when a labour which has begun under the most favourable circumstances is suffered to linger beyond fifty hours from the first *real* pain, it becomes a passive one, and is reduced either to a manual or instrumental labour. He includes those in which artificial aid in the extraction of the placenta was required, in the foregoing number of passive labours. In the eight instrumental labours, the forceps were employed in five instances; in one of these there were convulsions, and in two the head was firmly impacted in the pelvis, with the face to the pubis; in the other two the vertex presented.

The proportion of cases in which instruments were employed was one in eighty. This is a high proportion in comparison with the occurrences of private practice, or even the practice of lying-in hospitals; but the author explains it, by stating that there exists, particularly in the lower classes, a decided aversion amongst lying-in women against the interference of the accoucheur, which induces them to trust too much to time and nature, and to rely on them until nature becomes exhausted. Several cases, therefore, which, by more timely interference, would only have required manual assistance, now demanded instrumental. The more immediate superintendence of accouchers over the midwives in lying-in hospitals, enables them to restrain or prevent these occurrences, which cannot be easily effected where women are attended by midwives at their own homes. The same circumstances, only more widely extended, influence the whole of the puerperal state, and render the ratio of mortality much greater than that in private practice. Yet it is remarkable, that only four of the 640 are stated to have died after labour, and the whole of these after passive labour; which makes the proportion of deaths only 1 in 160, whilst the bills of mortality for London make it amount to 1 in about 109, as an average for the last few years. Of those four deaths, one happened in the woman attacked with convulsions; in another, the patient had been suffering from phthisis pulmonalis; the third, "the only patient (says the author) whom I have lost from peritonitis in the course of the twelve months, was that of a woman who had been delivered in the Brownlow-street Hospital, and occurred a few days after her confinement."

Some very curious and interesting statistical accounts of the ages of the patients then follow.

Of 623 pregnant women, whose ages could be ascertained, there were—

| Age. | Number. | Proportion. |
|---|---|---|
| From 16 to 20 .. | 7 | 1 in 89 |
| —— 20 to 30 .. | 325 | 1 in $1\frac{9}{10}$ |
| —— 30 to 40 .. | 244 | 1 in $2\frac{1}{4}$ |
| —— 40 to 50 .. | 46 | 1 in $13\frac{1}{2}$ |
| At 52.......... | 1 | 1 in 623 |
| Average age, 30 | Tot. 623 | *——— |

The collective age of these 623 women being 18,698 years.

* During the year 1816, some women were admitted into the *Maternité* as young as thirteen years of age, but none had applied who were older than forty. During the Revolution, one or two instances occurred of girls at eleven, and below that age, being received in a pregnant state into that Hospital. The number in 1816 stands thus:

| Age. | Number. | Proportion. |
|---|---|---|
| From 13 to 18.. | 75 | 1 in $35\frac{1}{10}$ |
| —— 18 to 30.. | 1905 | 1 in $1\frac{19}{50}$ |
| —— 30 to 40.. | 653 | 1 in $4\frac{57}{50}$ |
| Average age, 25 | Tot.2633 | |

"From this comparative statement, in the drawing-up of which I have employed the usual formulæ of arithmetical progression, it will be seen, 1st. That the age at which French women bear most children is between twenty-five and twenty-six; whereas, thirty appears to be the age in England; and 2dly. That English women are susceptible of bearing children to a much greater age than the French. In comparing, also, the number of women who became pregnant at an equal time of life in both countries, it is curious to observe, that the period between twenty and thirty years of age with us, and that of between eighteen and thirty in France, gives, very nearly indeed, the same proportion, namely, 1 in $1\frac{9}{10}$."

The report of one year is, the author remarks, too confined to admit of precise general inferences respecting these circumstances, so especially interesting to politicians and life-annuity societies; but a succession of these records, for several years, will furnish a good foundation for them.

The second section treats of *Abortion*. Here the author completely refutes the assertion of those who have stated that abortions but rarely happen amongst women of the lower ranks of life, except from accidental and external violence, at least as far as the women of this class in the metropolis are concerned. He has taken much pains in investigating the number of miscarriages which 400 married women had suffered within the last ten years, without including any circumstances relative to the pregnancy under which they applied to him at the Dispensary. Of those 400, 128 had miscarried at some period or other of their marriage, within the ten years, which makes a proportion of 1 in $3\frac{3}{25}$; the whole number of abortions, however, amounted to 305, giving a proportion of $2\frac{3}{10}$ for each woman. The same women produced, during the same term of years, 556 living children, at or near the full time; therefore the number of abortions was to that of children born at their full time as 18 to 32.

Some highly interesting observations follow on the probable causes of the abortions. This term the author thinks should be applied to birth at any period before the ninth month, not confined to any arbitrary time. He shows the impropriety of the division into *miscarriages*, and *premature labours* or *births*, according as the abortion takes place before or after the seventh month, by the fact that children have lived when produced before this period.

Dr. Granville refers the cases of abortion to two distinct classes: 1. Constitutional; 2d. Accidental. The constitutional are divided into (*a*) active, from local or general fulness, excessive irritability of the womb, increased local action; (*b*) passive, from local or general debility, certain morbid states of the body, defective organization, peculiarities of the ovum, and its situation in the womb, habit, and sympathy. The accidental are referred to fright, falls, violent exercise, violent passions, blows, incautious use of medicines, improper physical and moral treatment. Of the 305 cases noted in the Dispensary register, 156 appear to have been referable to the first, and 149 to the second class; and, from the care with which women generally retain in their memory all the circumstances relating to these accidents, it is probable that the specification is tolerably accurate.

A subdivision of this section treats of *the relative periods of pregnancy at which abortion takes place, and calculation of the probabilities of a woman miscarrying*, on the evidence presented in the cases already noticed. Of these, 185 occurred within the first three months of pregnancy, sixty-five from three to six months, and fifty-five from six to eight months. From which it appears, that, if a woman miscarries, the chances are, ten to sixteen that the abortion will take place at the third month or under, rather than during the fourth, fifth, or sixth month of pregnancy; ten to twenty-eight that it will take place between the third and sixth month, rather than at the seventh or eighth; ten to thirty-one that it will occur at the seventh or eighth month, rather than at any other period; and lastly, one to three that she will miscarry at all events.

On applying the same calculation to some returns made by the physicians of the *Société Maternelle* in Paris, the author has obtained nearly the same proportion with regard to the probabilities of miscarrying at certain given periods of pregnancy; but by no means the same number of abortions in reference to a given number of pregnancies, and during a determined period of time. The number in this respect being considerably smaller in Paris.

"To what cause this difference is to be ascribed," says the author, "I will not undertake to state, until I shall have seen more cases of this unfortunate occurrence among that class of people who seem most liable to it, and whom my frequent opportunities, when in the discharge of my duties at the Dispensary, afford me the means of watching and assisting.

"In many instances, I do not hesitate to say, this difference must necessarily arise from bad management during labour. It is no less lamentable than true, that, in many of the cases included in my calculation respecting those patients who have attended at the Dispensary, the poor women themselves have assigned the want of either skill or attention on the part of the midwife during labour, as the probable cause of their subsequent miscarriages; and I find, in fact, on inspection of the register, and on considering the cases of those who have fallen more particularly under my care since, that the miscarriages they have had subsequent to a previous labour, can readily be traced to such a morbid state of the parts as is likely to have been produced by unskilful management. Lacerations, prolapsi, and discharges of a bad character, announcing a diseased state of the womb, are unfortunately too common among the classes of women I am speaking of in this country. Now, in France, this cause, among the many others of abortion, can scarcely be taken into consideration, in accounting for the smaller number of miscarriages which occur in that country; for the regulations of the government with regard to females practising midwifery are so strict, and require such strong proof of preliminary as well as professional practical education, for the space of two years, at the national establishment of La Maternité, that no illiterate and uneducated woman is allowed ever to meddle with so important a branch of the medical profession, as a resource for failures in other business, or for want of something better to do."

The number of twin-cases in abortion, is also calculated by the author. Of the 128 women above alluded to, six miscarried of twins once; one, twice; and one, three times. The proportion of miscarriages of twins to that of single children, is then as 1 to 27. The author concludes this subject with a more particular account of the cases of abortion which occurred under his own observation in 1818. Of fourteen cases of threatened abortion from constitutional causes, to which the author was timely called, seven were prevented, although four of them had actually begun, and the flooding had lasted for some time. We agree with the author, that the doctrine of abortion is yet susceptible of much improvement; and we think that the sketch he has here presented of his views of this subject, promises much in the development of them which he gives us reason to expect from him.

The third section is on the Diseases of Females. Dr. Granville has formed a new arrangement of these diseases, which, excepting in one secondary and trivial point that will be presently specified, we think by far the best that has been proposed, as it comprises all the perspicuity that is desirable for elementary instruction, and presents such general views as are qualified to guide, as far as such classifications can do, the conduct of the medical practitioner. His three leading divisions are, diseases by which the female sex is more especially affected during the age of puberty; the age of propagation; the critical age. The diseases of the age of puberty are distinguished into 'organic,' 'constitutional,' 'secretory.' The *organic* diseases are again subdivided into 'congenital,' 'subsequent.' The *constitutional*, into 'those preceding menstruation,' 'attending menstruation.' The *secretory*, into 'those from blood-vessels,' 'from surfaces.' It is this subdivision which we think is of doubtful propriety; for none of the secretions can be strictly said to come *immediately* from blood-vessels, whilst they might all be comprised in the view of secretion from surfaces. The author, too, comprises in it affections which are not properly morbid states of the secretory functions, as *hemorrhage: menorrhagia* is, probably, liable to the same objection. This inconvenience might have been obviated, if he had taken the peculiar functions of the several organs as the base of this subdivision, instead of arranging the diseases it should comprise as secretory diseases; and the alteration here proposed would coincide very well with the other two genera of organic and constitutional affections.

The diseases of the age of propagation, or, more properly speaking, those which especially require the attention of the physician during this epoch, are first distinguished, in the same way as the preceding class, into organic, constitutional, and secretory. The first is subdivided into those impeding propagation, and those often consequent on propagation. The constitutional, into those occurring during pregnancy, and those after pregnancy.

The organic diseases of the critical age, are subdivided into those attended by discharges, and those not essentially attended by discharges; and the constitutional affections, into inflammatory, nervous, muscular.

Our limits will not permit us to adduce the author's specification of particular diseases according to the above classification; but we do not much regret this restriction; for we consider that the more studious part of the profession will peruse the original.

A general table of the cases treated at the Dispensary during the last year, with some abstract views of them, and several good particular clinical remarks, then follow. Of twenty cases of "peritonitis puerperarum," which the author regards as the true *puerperal fever*, but one died; and in this instance the assistance of the author was not solicited until a late period of the disease, "when serous effusion in the cavity of the abdomen had taken place, and when bleeding, the heroic remedy for this disease, was no longer of service." This malady, Dr. Granville says, is often *endemic* in certain hospitals; and the physical constitution of the atmosphere may also render it *epidemic;* but he does not think that it is ever *contagious*, "according to the purest and most exact knowledge of the principles of contagion." Of eleven cases of leucorrhœa, eight were completely cured, and one greatly relieved. Local applications and strengthening medicines formed the principal remedial measures.

The diseases of children are considered in the fourth section. These are arranged into *infantiles*, beginning at the term of birth, and ending at the period of the first dentition, which is most commonly also that of weaning; *puériles*, including the time which elapses between the first and second dentition; and *incertæ ætatis*, which embraces several, *acute* and *chronic*, occurring from birth to the age of puberty. After a brief sketch of the miserable state of a great proportion of the poor of the metropolis, and some remarks on the influence of this on the diseases and mortality of their children, the author gives an account of the cases coming within his practice at the Dispensary during 1818, similar to that already noticed respecting the diseases of women.

The second part of this work is devoted to histories of interesting cases, illustrated by clinical remarks; which we are absolutely prevented from taking into particular consideration, by the impossibility of giving any thing like a sufficient account of them in an abstract at all conformable with the limits of this Journal. We recommend them to the attention of our readers; and we are inclined to believe, that the outline we have here given of this work will present indications of the merit of the whole, sufficient to render this recommendation generally effectual.

---

